# Wilms’ tumor 1-associating protein promotes renal cell carcinoma proliferation by regulating CDK2 mRNA stability

**DOI:** 10.1186/s13046-018-0706-6

**Published:** 2018-02-27

**Authors:** Jingyuan Tang, Feng Wang, Gong Cheng, Shuhui Si, Xi Sun, Jie Han, Hao Yu, Wei Zhang, Qiang Lv, Ji-Fu Wei, Haiwei Yang

**Affiliations:** 1 Department of Urology, The First Affiliated Hospital of Nanjing Medical University, Nanjing, 210029 China; 2 Department of Urology, Jiangsu Province Hospital of TCM, Affiliated Hospital of Nanjing University of TCM, Nanjing, 210029 China; 3 Research Division of Clinical Pharmacology, The First Affiliated Hospital of Nanjing Medical University, Nanjing, 210029 China; 4 Jiangsu Breast Disease Center, The First Affiliated Hospital of Nanjing Medical University, 300 Guangzhou Road, Nanjing, 210029 China

**Keywords:** WTAP, CDK2, Renal cell carcinoma, Prognosis, Proliferation

## Abstract

**Background:**

Wilms’ tumor 1-associating protein (WTAP) plays an important role in physiological processes and the development of tumor such as cell cycle regulation. The regulation of cell cycle is mainly dependent on cyclins and cyclin-dependent protein kinases (CDKs). Recent studies have shown that CDKs are closely related to the tumor diagnosis, progression and response to treatment. However, their specific biological roles and related mechanism in renal cell carcinoma (RCC) remain unknown.

**Methods:**

Quantitative real-time PCR, western blotting and immunohistochemistry were used to detect the expression of WTAP and CDK2. The survival analysis was adopted to explore the association between WTAP expression and the prognosis of RCC. Cells were stably transfected with lentivirus approach and cell proliferation and cell cycle, as well as tumorigenesis in nude mice were performed to assess the effect of WTAP in RCC. RNA immunoprecipitation, Luciferase reporter assay and siRNA were employed to identify the direct binding sites of WTAP with CDK2 transcript. Colony formation assay was conducted to confirm the function of CDK2 in WTAP-induced growth promoting.

**Results:**

In RCC cell lines and tissues, WTAP was significantly over-expressed. Compared with patients with low expression of WTAP, patients with high expression of WTAP had lower overall survival rate. Additionally, cell function test indicated that cell proliferation abilities in WTAP over-expressed group were enhanced, while WTAP knockdown showed the opposite results. Subcutaneous xenograft tumor model displayed that knockdown of WTAP could impede tumorigenesis in vivo. Mechanism study exhibited that CDK2 expression was positively associated with the expression of WTAP. Moreover, WTAP stabilized CDK2 transcript to enhance CDK2 expression via binding to 3′-UTR of CDK2 transcript. Additionally, specific inhibitors of CDK2 activity and small interfering RNA (siRNA) of CDK2 expression inhibited WTAP-mediated promotion of proliferation.

**Conclusions:**

These findings suggest that WTAP may have an oncogenic role in RCC through physically binding to CDK2 transcript and enhancing its transcript stability which might provide new insights into RCC therapy.

**Electronic supplementary material:**

The online version of this article (10.1186/s13046-018-0706-6) contains supplementary material, which is available to authorized users.

## Background

Renal cell carcinoma (RCC) accounts for approximately 5% of all adult malignant neoplasms and is among the top 10 most common cancer in both men and women. In 2017, it is estimated that 63,990 new cases and 14,400 deaths will be recorded in the United States [1]. Surgery remains the mainstay treatment for patients with a localized stage because RCC is relatively resistant to radiotherapy [2] and chemotherapy [3]. However, approximately 30% of patients have locally advanced or developed metastases at the time of diagnosis and about 30–50% of patients will develop metastatic RCC following surgical resection of the primary tumor [4]. These patients with metastatic RCC are inoperable and the long-term prognosis remains poor though recent therapeutic developments, such as molecular targeted therapy, have improved the overall survival [5]. Therefore, understanding the detailed molecular mechanism of cancer progression is crucial for the high prevalence of the cancer and the development of effective interventions of RCC.

Wilms’ tumor 1 (WT1) gene was originally discovered as a tumor suppressor gene inactivated in a subset (∼15%) of pediatric renal cancers unrelated to RCC known as Wilms’ tumors [6]. Also, WT1 can act as a tumor suppressor in RCC via multiple pathways leading to down-regulation of human telomerase reverse transcriptase (hTERT) [7]. Later, a human Wilms’ tumor 1-associating protein, WTAP was isolated by the yeast two-hybrid system. Like WT1, WTAP is a nuclear protein and localizes throughout the nucleoplasm as well as in speckles and partially co-localizes with splicing factors [8]. WTAP is widely expressed in various tissues and plays an important role in the normal cellular and physiological processes, such as cell cycle regulation [9], RNA alternative splicing [10], m6A methylation modification [11], X-chromosome inactivation [12], eye development [13], and regulating the balance between quiescence and proliferation [14]. WTAP played an oncogenic role in many tumors including cholangiocarcinoma [15], glioblastoma [16] and acute myeloid leukemia [17]. Moreover, its overexpression was correlated with a poor prognosis in acute myeloid leukemia [17], malignant glioma [18], and pancreatic ductal adenocarcinoma [19]. In the investigation for the oncogenic mechanism of WTAP, it was found that WTAP could act as an oncogenic protein by regulating the expressions of matrix metalloproteinase (MMP) 7, MMP28, cathepsin H and Muc1 [15], controlling epidermal growth factor signaling [16], regulating mTOR pathway and targeting the WT1-TBL1 axis [20]. However, unlike WT1, the role of WTAP in RCC was still unknown.

In cancer, proliferation is mostly driven by altered cell cycle progression [21]. The regulation of cell cycle is mainly dependent on cyclins and cyclin-dependent protein kinases (CDKs). Recent studies have shown that cyclins and CDKs are exceptionally expressed in many tumors, closely related to the tumor diagnosis, progression and response to treatment [22]. In normal cells, WTAP was an important factor in the regulation of cell cycle by affecting cyclinA2 mRNA stability [23]. It was also a putative splicing regulator that is supposed to be an essential factor for cell cycle progression [9]. It needs further investigation whether WTAP exhibited its oncogenic role by regulating the cell cycles proteins such as cyclins and CDKs.

In the present study, we investigated the role of WTAP in RCC related mechanisms. We found that 1. WTAP expression was up-regulated in RCC tissues and high expression of WTAP in RCC was related to poor prognosis. 2. WTAP could promote the RCC cell proliferation in vitro and in vivo. 3. WTAP could promote the RCC cell proliferation by regulating CDK2 mRNA stability. Therefore, WTAP may act as a novel diagnosis and prognostic biomarker for RCC patients.

## Methods

### Clinical specimens

RCC and adjacent non-cancerous tissues were obtained from patients who underwent radical nephrectomy or partial nephrectomy at the Department of Urology of the First Affiliated Hospital of Nanjing Medical University from February 2008 to January 2010. The follow-up deadline was January 2016. All patients provided appropriate informed consent and the study was approved by the Local Ethics Committees of the First Affiliated Hospital of Nanjing Medical University.

### Tissue microarray (TMA) and immunohistochemistry (IHC)

TMA was constructed using 85 cases of formalin-fixed, paraffin-embedded RCC samples. IHC was performed on TMA to evaluate WTAP and CDK2 protein expression. The tissue samples were stained with the following primary antibodies respectively: anti-WTAP antibody (Abcam, USA) and anti-CDK2 antibody (Cell Signaling Technology, USA). Standard staining protocols were used [24]. Stained tissues were scored for staining intensity (SI) and the percentage of positive cells (PP). SI was scored on a scale of 0 to 3 (0, negative staining; 1, weak staining; 2, moderate staining; 3, strong staining) and PP was scored into five categories: 0 (0% positive cells), 1 (< 10%), 2 (11% to 50%), 3 (51% to 80%) or 4 (> 80%). The final staining score was calculated by multiplying SI and PP score, resulting in a score value ranging from 0 to 12. The positive level of immunohistochemical staining was scored by two urologists and patients with different scores were divided into low- (0–7) and high-staining (8–12) groups.

### Cell culture

Human RCC cell lines were purchased from the American Type Culture Collection (ATCC, USA) and cultured in McCoy’s 5A medium (Gibco, USA) or in DMEM media (Gibco, USA) containing 10% fetal bovine serum (Gibco, USA) and 1% penicillin/streptomycin (Invitrogen, USA) in humidified air at 37 °C with 5% CO2.

### Transfection

Lentivirus constructing of WTAP knockdown or overexpression were obtained from OBIO (Obio Technology Corp, China). Cells were plated in 6 wells dishes at 50% confluence and infected with WTAP overexpression lentivirus (termed as WTAP), a negative control (termed as NC), WTAP knockdown lentivirus (termed as shWTAP1, shWTAP2), and a scramble control (termed as shNC) in Caki-1 and ACHN cells. Pools of stable transductions were generated by selection using puromycin (4 μg/ml) for 2 weeks.

CDK2 siRNA (5′-GUACGGAGUUGUGUACAAATT-3′) and negative control was obtained from GenePharma (GenePharma, China). Transfections were performed using the Lipofectamine 2000 kit (Invitrogen, USA) according to the manufacturer’s instructions.

### RNA isolation and qRT-PCR

Total RNA was extracted from clinical samples or cultured cell lines by Trizol reagent (Invitrogen, USA) and used to synthesize cDNA with the Primescript RT Reagent (TaKaRa, Japan) according to the manufacturer’s instructions. For mRNA analysis, all qRT-PCR was performed with SYBR® Premix Ex Taq™ Reagent (TaKaRa, Japan) by using StepOne Plus Real-Time PCR system (Applied Biosystems, USA). The following primers were used for qRT-PCR:

WTAP, Forward: 5′-CTTCCCAAGAAGGTTCGATTGA-3′.

Reverse: 5′- TCAGACTCTCTTAGGCCAGTTAC-3′.

CDK2, Forward: 5′-CCAGGAGTTACTTCTATGCCTGA-3′.

Reverse: 5′- TTCATCCAGGGGAGGTACAAC-3′.

cyclin A2, Forward: 5′-TTATTGCTGGAGCTGCCTTT-3′.

Reverse: 5′-CTCTGGTGGGTTGAGGAGAG-3′.

β-actin, Forward: 5′-CCTGGCACCCAGCACAAT-3′.

Reverse: 5′-GCTGATCCACATCTGCTGGAA-3′.

GADPH, Forward: 5′- CCCAGCCTCAAGATCATCAGCAATG-3′.

Reverse: 5′-ATGGACTGT GGTCATGAGTCCTT-3′.

Fold changes in mRNA expression were calculated using 2^-ΔΔCt^ method and normalized based on β-actin with ABI Step One Software version 2.1.

### RNA stability

Caki-1 and ACHN cells transfected with the control lentivirus, WTAP lentivirus or WTAP knockdown lentivirus were treated with 5μg/ml actinomyclin D (Act D) for 0, 1, 2, 4, 6, 8 h. Total RNAs were harvested, and then subjected to quantitative RT-PCR analysis. The level of CDK2 or cyclin A2 transcript was normalized to that of β-actin or GADPH control and the relative half-life of CDK2 or cylcin A2 was calculated.

### Protein isolation and western blot

Total cellular proteins were lysed by RIPA buffer containing protease inhibitors (Sigma, USA). The protein concentration was quantified using a BCA Protein Assay kit (Pierce, USA). Total protein was separated using 10% SDS-PAGE gel and transferred to a PVDF membrane (Millipore, USA). Western blot analysis followed a standard procedure. After incubated with the primary antibodies anti-WTAP, anti-CDK2 or anti-GAPDH (Cell Signaling Technology, USA), the membranes were then incubated with peroxidase (HRP)-conjugated secondary antibodies (Cell Signaling Technology, USA). After washes, signals were detected using a chemiluminescence system (Bio-Rad, USA) and analyzed using Image Lab Software.

### Cell proliferation assay

Cell proliferation was assessed using a cell counting kit-8 assay (CCK8, Dojindo, Japan). Pretreated cells were seeded into a 96-well plate with 3 × 10^3^ cells/well and cultured for 1, 2, 3, and 4 days. In inhibitor experiment, SU9516 and K03861 (MCE, USA) were added into the wells at the concentration of 20 μM and 10 μM respectively after cell attachment. The absorbance was measured at 450 nm with a microplate reader after incubated at 37 °C for 3 h.

### Colony formation assay

Pretreated cells were seeded into 6-well plates (600 cells/well). After incubation for 24 h, the cells were incubated for a further two weeks in the absence or presence CDK2 inhibitors (20 μM SU9516 or 10 μM K03861). The colonies were fixed in paraform for 30 min, washed with PBS and stained with 0.1% crystal violet.

### Cell cycle assay

1 × 10^6^ cells were collected, washed with PBS and fixed with 75% cold ethanol for 24 h at − 20 °C. After washed and stained with propidium iodide (BD, USA) at room temperature for 30 min, cells were assessed by flow cy4ometry (Becton Dickinson, USA).

### Transwell cell migration and invasion assay

In this assay, transfected cells were seeded into the upper chambers of each transwell, which was coated with or without Matrigel (BD Biosciences, USA) for the invasion and migration assays. Medium containing 10% FBS was added to the bottom chamber. After incubation at 37 °C for 48 h, cells on the surface of the upper membrane were removed carefully, while cells adhered to the lower membrane were fixed in methanol for 20 min and stained with 0.1% crystal violet for 20 min. Migratory and invasive cells were counted on an inverted microscope in five randomly fields and experiment was repeated three times.

### Xenograft experiments in vivo

Animal studies were approved by the Animal Research Ethics Committee of Nanjing Medical University. The female BALB/c nude mice (5 weeks old) were obtained from the Model Animal Research Center of Nanjing University. ACHN cells stably transfected with either the WTAP knockdown lentiviral vector or control vector were injected subcutaneously into the flank of each mouse. Tumor size was measured every five days by the following formula: Volume = length×width^2^ × 0.52. The mice were sacrifced at 30th day after seeding the tumor cells and tumors were removed, weighed, fixed, and embedded for IHC.

### Dual-luciferase reporter assay

Dual-luciferase reporter assay was performed, in triplicate, according to manufacturer′s instructions (Promega, USA). The full-length CDK2 3′-UTR was inserted into the pLenti-UTR-Luc vector (Promega, USA) between EcoRI and XhoI sites. Renilla luciferase vector (pRL-CMV; Promega, USA) used as intermal control, and a pGL3 reporter which contained of CDK2 3′-UTR were transfected into Caki-1 and ACHN WTAP overexpression cells (WTAP) and the control cells (NC) using Lipofectamine 3000 reagent (Invitrogen USA). Next, cells were harvested and luciferase activity was measured at 48 h, according to manufacturer′s procedure. The fold change in relative luciferase activity was a ratio of the luciferase activity induced by WTAP divided by that induced by NC.

### RNA immunoprecipitation (RIP)

RIP experiments were performed using Magna RIP RNA-Binding Protein Immunoprecipitation Kit (Millipore, USA) according to the manufacturer’s instructions. Briefly, RCC cells were lysed with RIP lysis buffer. Cell lysates were immunoprecipitated with anti-WTAP antibody or non-immunized IgG at 4 °C overnight, followed by RNA purification. After this step, RT-PCR and qRT-PCR were used to measure the levels of CDK2 transcript in the WTAP or IgG immunocomplexes.

### Statistical analysis

Statistical analysis was performed using SPSS 22.0 software. The results are presented as the mean ± SD from three different independent experiments. The differences between groups were analyzed by Student’s t test and the χ2 test was used to analysis the differences between categorical variables. The survival curves were drawn using the Kaplan–Meier method and compared by the log-rank test. *P* < 0.05 was considered statistically significant.

## Results

### WTAP is significantly upregulated in RCC tissues and related to RCC patient prognosis

**Fig. 1:**
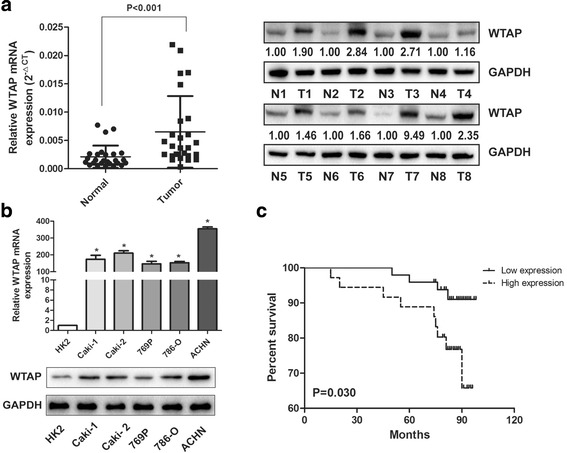
Up-regulation of WTAP is associated with proliferation and served as a prognostic factor in RCC. **a** WTAP mRNA and protein expression in the pairs of RCC tissues and matched adjacent tissues. The fold change of WTAP is shown below each lane. **b** WTAP mRNA and protein expression in RCC cell lines and normal epithelium cell of renal tubule HK2. (C) Kaplan-Meier survival curves of overall survival in 86 RCC patients based on WTAP IHC stains. The log-rank test was used to compare differences between two groups

To explore the potential role of WTAP in RCC, we first examined the mRNA and protein expression of WTAP in tumor tissues and paired adjacent tissues by qRT-PCR and western blot. We found that WTAP was significantly up-regulated in RCC tissues compared with the adjacent tissues (Fig. 1a). We also validated the expression of WTAP in five RCC cell lines and found WTAP was also up-regulated compared with the normal epithelium cell of renal tubule HK2 (Fig. 1b). To further explore the relationship between WTAP expression and clinicopathologic features, we performed IHC on 85 RCC patient samples with long-term clinical follow-up. Subsequent analysis revealed that WTAP expression distinctly associated with tumor size and TNM stage (Table 1). The high expression group had larger tumor size and higher TNM stage. Furthermore, Kaplan-Meier survival curves showed patients with high WTAP expression had a worse prognosis and poorer overall survival rate compared those with low WTAP expression (Fig. 1c), which was consistent with the results from tumor samples with detailed clinical information which were downloaded from TCGA database (https://cancergenome.nih.gov) (Additional file 1: Fig. S1). Thus, we proposed that the high WTAP expression might be important during RCC development and progression and could be a potential prognostic marker for RCC patients.

**Table 1:** Association of WTAP expression with clinicopathologic characteristics of the renal cancer patients

Parameters	Number of cases	WTAP expression		*P*-value
		Low (%)	High (%)	
Age (years)	60	36(60)	24(40)	0.496
< 60	25	13(52)	12(48)	
≥ 60				
Gender				0.244
Male	51	32(62.7)	19(37.3)	
Female	34	17(50)	17(50)	
Tumor size (cm)				0.015^a^
≤ 4	39	28(71.8)	11(28.2)	
> 4	46	21(45.7)	25(54.3)	
Histological grade				0.22
I-II	71	43(60.6)	28(39.4)	
III-IV	14	6(42.9)	8(57.1)	
TNM stage				0.002^a^
I	62	42(67.7)	20(32.3)	
II-IV	23	7(30.4)	16(69.6)	
CDK2				0.004^a^
Negative	34	26(76.5)	8(23.5)	
Positive	51	23(45.1)	28(54.9)	

^a^Statistically significant

### WTAP promotes RCC cell proliferation and migration in vitro

According to the significant association between the expression of WTAP and the tumor size as well as TNM stage, we further investigated the role of WTAP in RCC cell proliferation and migration in vitro. Firstly, the expression levels of WTAP were confirmed by qRT-PCR and western blot (Additional file 2: Fig. S2 A and B).

**Fig. 2:**
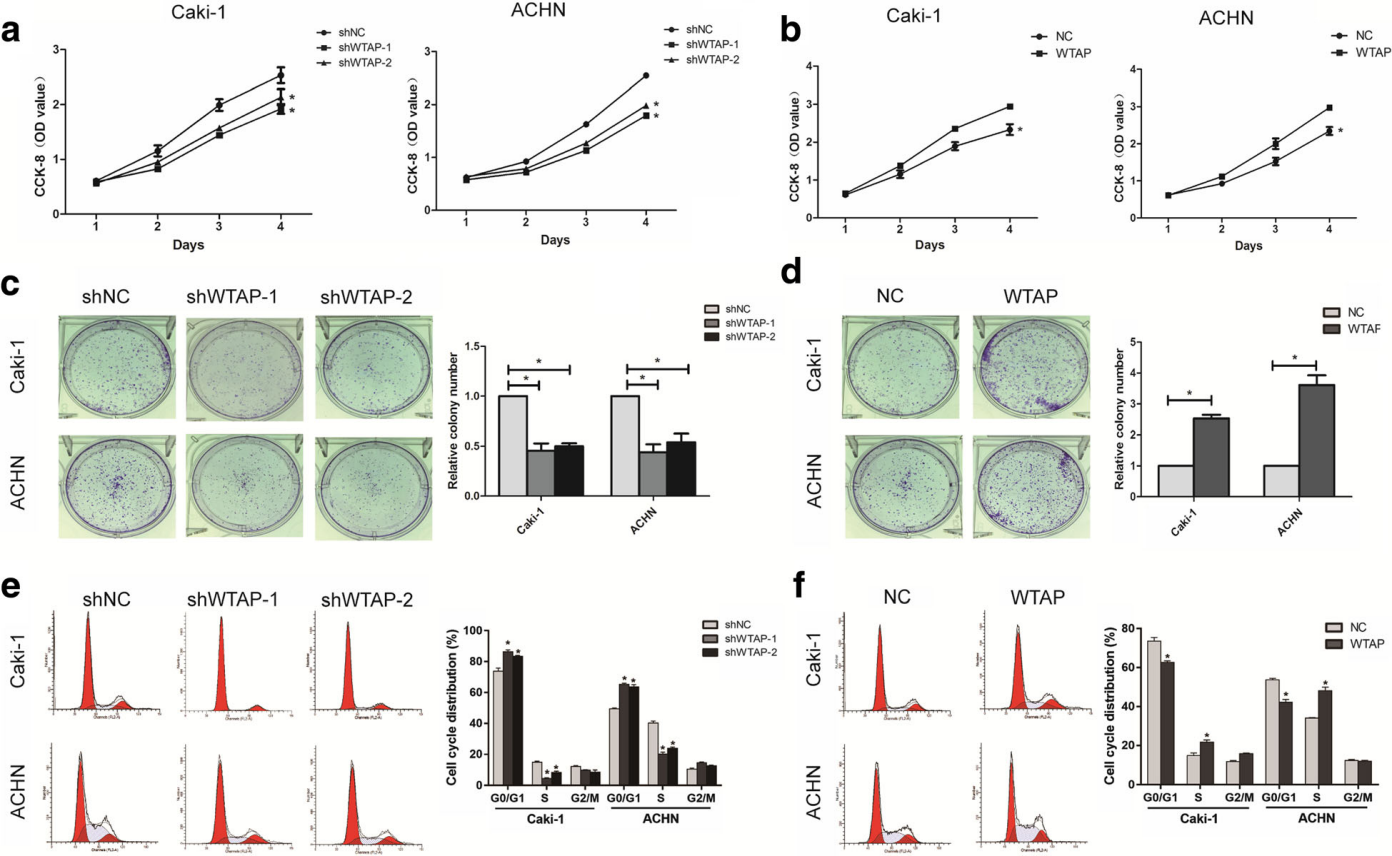
WTAP promotes RCC cell proliferation in vitro. **a**, **b)** Proliferation of RCC cells assessed by CCK8 assays. **c**, **d)** The efficiencies of cell colony formation in Caki-1 and ACHN cells with WTAP knockdown or overexpression. (**e**, **f** Cell cycle analyzed by flow cytometry of Caki-1 and ACHN cells after the knockdown or overexpression of WTAP. The histogram indicates the percentage of cells in G0/G1, S and G2/M. Data represent the mean ± SD from three independent experiments, *P < 0.05

Next, CCK8 assay indicated that cell proliferation was inhibited by WTAP knockdown (Fig. 2a), whereas enhanced cell growth occurred after WTAP overexpression compared with the NC group (Fig. 2b). WTAP overexpression also could promote cell proliferation in normal cell HK-2 (Additional file 3: Fig. S5). In colony formation assays, knockdown of WTAP could significantly decrease cell colony formation efficiency (Fig. 2c), yet the colony formation rate was increased in WTAP up-regulated cells (Fig. 2d). Moreover, cell cycle analysis used by flow cytometry demonstrated an increased percentage of G1 phase in WTAP knockdown cells (Fig. 2e) while WTAP overexpression had opposite effects (Fig. 2f). These results suggested that WTAP could promote the proliferation of RCC cells via accelerating the cell cycle progression.

These data together suggested that WTAP may be involved in RCC proliferation, migration and invasion (Additional file 4: Fig. S3), acting as a positive regulator.

### WTAP promotes tumorigenesis in vivo

**Fig. 3:**
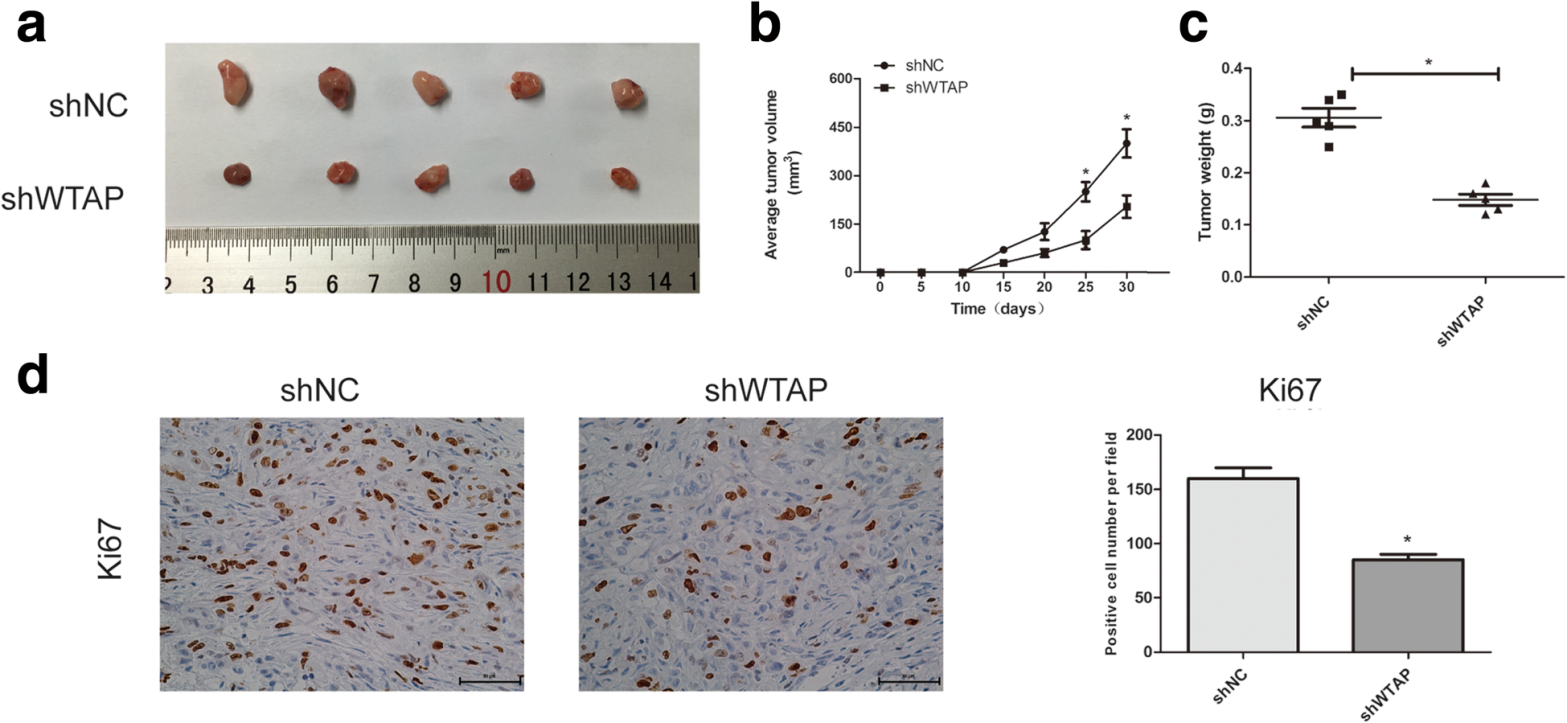
WTAP promotes tumorigenesis in vivo. **a** Subcutaneous tumor model of ACHN cells with WTAP knockdown. **b**, **c)** Tumor volume and weight were measured at the indicated weeks after mice were transplanted. **d** IHC analysis of ki-67 in xenografs. The histogram indicates the ki-67 positive cells from panel. Data represent the mean ± SD from three independent experiments,*P < 0.05

To further study the effects of WTAP on tumor growth in vivo, we established subcutaneous xenograft tumor model using WTAP-knockdown or the control cells. As expected, tumor grew dramatically slower in WTAP-knockdown group (shWTAP) compared with the control group (shNC) (Fig. 3a and b). Similar results were observed in the mean tumor weight (Fig. 3c). Furthermore, we found a significant decrease in the positive rate of Ki67 in WTAP-knockdown group by IHC analysis (Fig. 3d). Overall, these results demonstrated that WTAP silencing could inhibit tumorigenesis in vivo.

### WTAP regulated CDK2 expression in RCC cells and correlated with CDK2 expression in human RCC tissues

**Fig. 4:**
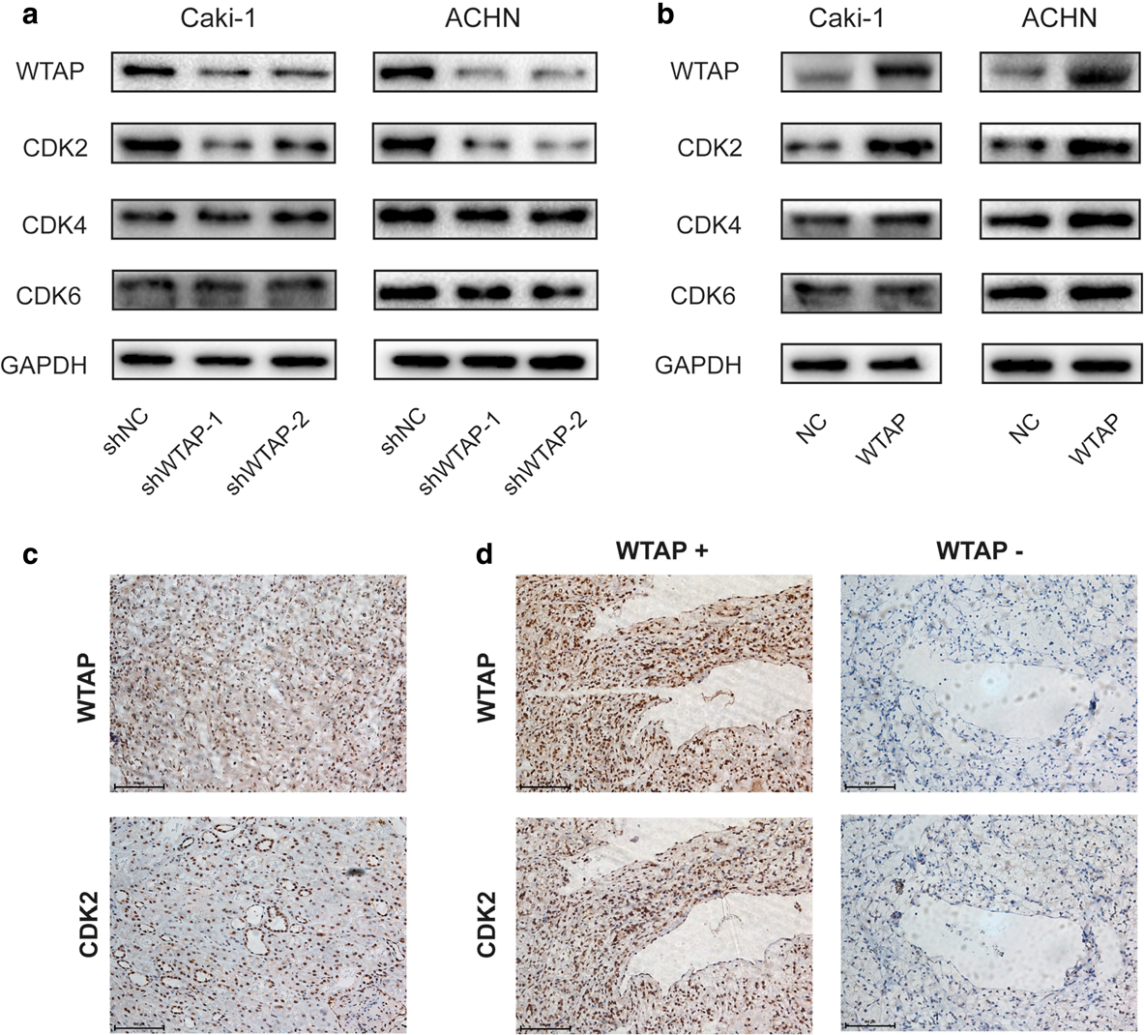
WTAP regulated CDK2 expression in RCC cells and correlated with CDK2 expression in human RCC tissues. **a**, **b)** Western blot analysis of CDK2, CDK4 and CDK6 expression in Caki-1 and ACHN cells with WTAP knockdown or overexpression. CDK2 expression was obviously decreased in WTAP-knockdown cells whereas increased in WTAP overexpression cells. **c** IHC analysis of WTAP and CDK2 in RCC tissue microarray at 200× magnifcation. WTAP and CDK2 were both mainly expressed in the nucleus. **d** WTAP positive RCC expressed high level of CDK2; WTAP negative RCC expressed low level of CDK2. Scale bars indicate 100 μm

To investigate how WTAP regulated the cell proliferation of RCC cancer cells, we used western blot to investigate the expression change of cell cycle related protein CDK2, CDK4 and CDK6 by the knockdown or overexpression of WTAP. We found that the knockdown of WTAP could prominently decrease CDK2 expression (Fig. 4a), whereas WTAP overexpression increasing CDK2 expression compared with the control group (Fig. 4b). It indicated that WTAP could regulate CDK2 expression in RCC cancer cells.

Furthermore, we investigated the expression of WTAP and CDK2 in TMA. Both WTAP and CDK2 were mainly expressed in the nucleus (Fig. 4c). Moreover, the representative images of CDK2 expression in WTAP high expression and low expression RCC cancer tissues were displayed (Fig. 4d), and it showed that CDK2 expression was positively correlated with WTAP expression in RCC cancer (Table 1), which was consistent with the results from TCGA database (Additional file 5: Fig. S4.). Together, we found that WTAP could regulate CDK2 expression in RCC cells and correlate with CDK2 expression in human RCC tissues.

### WTAP enhanced the stability of the CDK2 mRNA by directly binding to CDK2 transcript

**Fig. 5:**
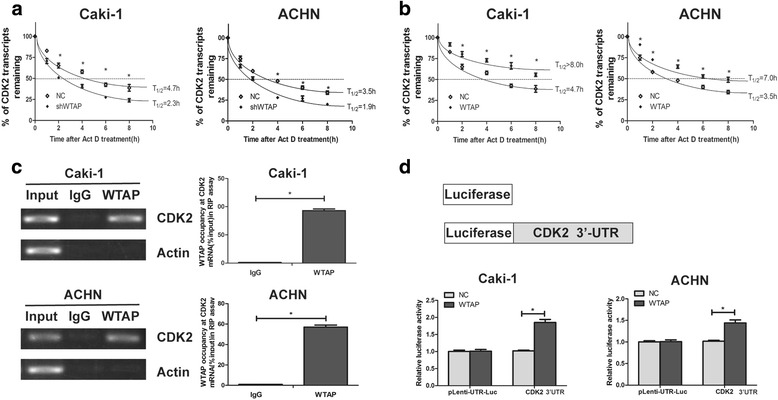
WTAP directly bound to CDK2 transcript and regulated its mRNA stablility. **a** Knockdown of WTAP could shorten the half-life of CDK2 transcript. **b** Ectopic expression of WTAP could lengthen the half-life of CDK2 transcript. **c** WTAP could bind to CDK2 transcript in vivo in RCC cells. RCC cell lysates were immunoprecipitated with WTAP antibody or control IgG followed by RT-PCR and qRT-PCR. **d** The luciferase activity for the reporter carrying CDK2 3′-UTR was increased by WTAP overexpression. Caki-1 and ACHN cells with WTAP overexpression and the control were transfected with pGL3 control reporter or pGL3 reporter carrying CDK2 3′-UTR. Data represent the mean ± SD from three independent experiments,*P < 0.05

To explore whether WTAP could effect on the transcript levels of CDK2, we treated the WTAP knockdown or overexpression cells with actinomyclin D (Act D). We found that WTAP knockdown significantly decreased the relative half-life of CDK2 transcript (Fig. 5a). The relative half-life for CDK2 mRNA was decreased from 4.7 h to 2.3 h in Caki-1 cells, and the relative half-life for CDK2 mRNA was decreased from 3.5 h to 1.9 h in ACHN cells. Furthermore, the half-life of CDK2 transcript was increased by overexpression of WTAP in both Caki-1 and ACHN cell lines (Fig. 5b). The relative half-life for CDK2 mRNA was increased from 4.7 h to more than 8.0 h in Caki-1 cells, and the relative half-life for CDK2 mRNA was increased from 3.5 h to 7.0 h in ACHN cells. These data suggested that WTAP could increase the stability of CDK2 mRNA.

In our study, we also found that the knockdown of WTAP could prominently decrease cyclin A2 expression, whereas WTAP overexpression increasing cyclin A2 expression compared with the control group in Caki-1 cell line (Additional file 6: Fig. S 6A). Next, we found the expression of WTAP and cyclin A2 was positively correlated in RCC tissues which were downloaded from TCGA database (https://cancergenome.nih.gov/) (Additional file 6: Fig. S 6B) (2-tailed Spearman’s correction, R = 0.25, *P* = 1.3e-12). Furthermore, we also found WTAP knockdown significantly decreased the relative half-life of cyclin A2 transcript (Additional file 6: Fig. S 6C). However, the half-life of cyclin A2 transcript was increased by overexpression of WTAP in Caki-1 cell line (Additional file 6: Fig. S 6C). These data suggested that WTAP could also increase the stability of cyclin A2 transcript. Our results were consistent with the former study which reported WTAP could affect cyclin A2 mRNA stability in normal cell [23].

Next, we performed RNA immunoprecipitation (RIP) to validate whether WTAP could bind to CDK2 transcript. RCC cells were immunoprecipitated with WTAP antibody or control rabbit IgG. RT-PCR and qRT-PCR analysis demonstrated that CDK2 transcript was significantly enriched in the WTAP immunocomplexes, but not in the control IgG immunocomplexes (Fig. 5c). As a negative control, β-actin transcript was unable to detectable in WTAP immunocomplexes and IgG immunocomplexes. It demonstrated that WTAP could physically bind to CDK2 transcript. Finally, we proved that WTAP could bind to CDK2 mRNA in vivo and stabilize the CDK2 transcript.

Finally, to verify that CDK2 3′-UTR were required for WTAP increasing CDK2 expression, we performed a dual-luciferase assay using pLenti-UTR-Luc reporters that carried CDK2 3′-UTR or empty vector in Caki-1 and ACHN WTAP overexpression cells and the control cells. Overexpression of WTAP significantly increased the luciferase activity of the CDK2 3′-UTR reporter vector but not of the empty vector (Fig. 5d). Taken together, these data indicated that WTAP could bind to CDK2 3′-UTR.

Additionally, in order to confirm the result, we used GADPH as another more stable reference mRNA to explore whether WTAP could effect on the stability of the CDK2 transcript. The result was consistent with the former one (Additional file 7: Fig. S 7).

### CDK2 interference decreased the proliferation promoting induced by WTAP in RCC cells

**Fig. 6:**
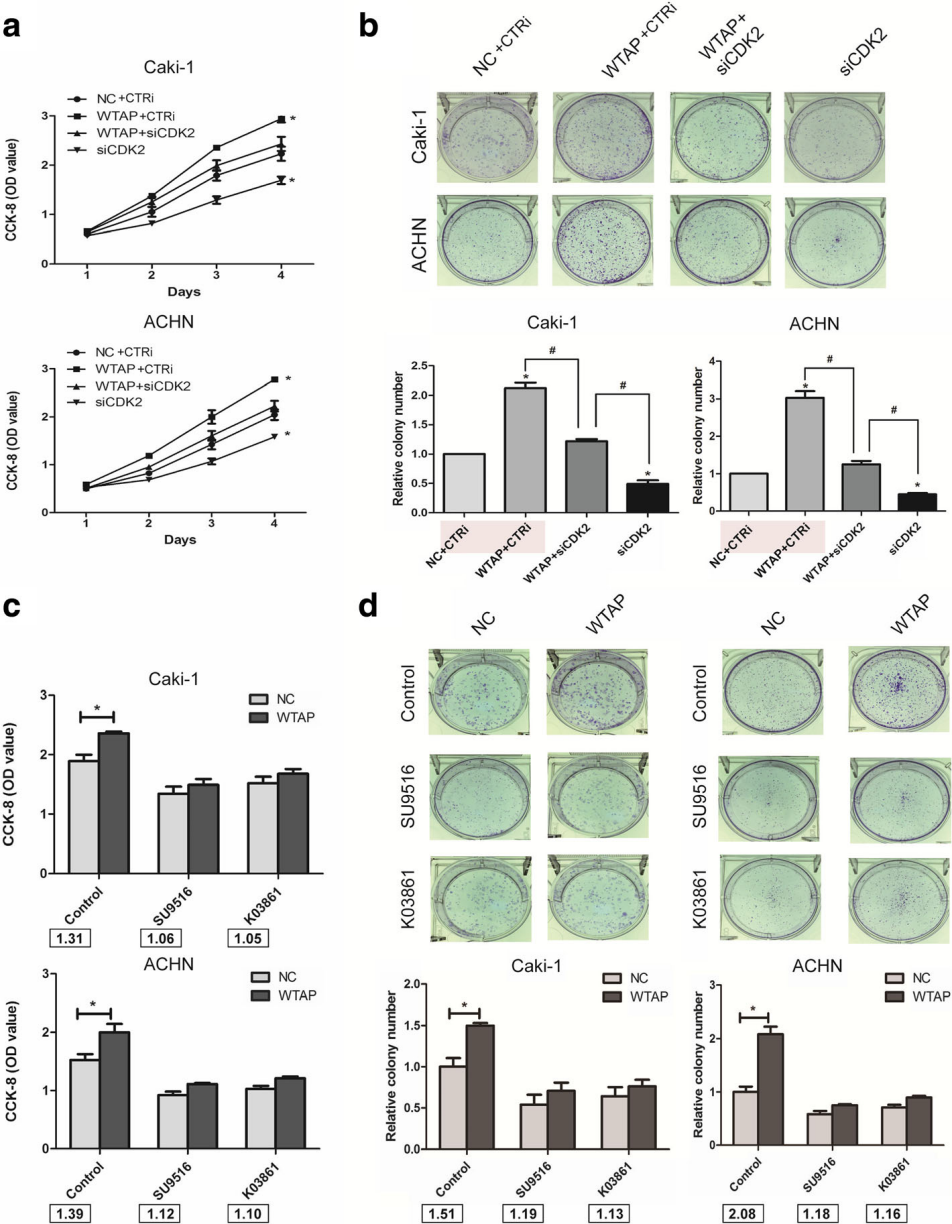
Reduced of CDK2 expression suppressed the cell proliferation induced by WTAP in RCC cells. **a**, **b)** CCK8 and clone formation assays were used to measure the effect of CDK2 small interference RNA (siCDK2) on Caki-1 and ACHN cells with WTAP overexpression. **c**, **d)** The effect of CDK2 inhibitors (SU9516 and K03861) on Caki-1 and ACHN cells with WTAP overexpression were measure by CCK8 and clone formation assays. The fold change (NC/WTAP) was stated under the histogram. Data represent the mean ± SD from three independent experiments,*P < 0.05

To confirm the contribution of CDK2 to the WTAP-regulated cell proliferation, we transfected WTAP overexpression cells and the control cells with CDK2 small interference RNA (siCDK2) or a control (CTRi) into Caki-1 and ACHN cell lines. We found that CDK2 knockdown resulted in a decreased cell growth by CCK8 assay. In addition, CDK2 knockdown totally reversed the proliferation induced by WTAP in RCC cells (Fig. 6a). The clone formation assays showed the similar results (Fig. 6b).

Moreover, we chose two selective CDK2 inhibitors SU9516 and K03861 to further explore if WTAP drives cell proliferation through a CDK2-dependent mechanism. The WTAP overexpressed (WTAP) and the control cells (NC) were treated with 20 uM SU9516, 10 uM K03861 respectively. We found that the OD value fold (NC/WTAP) and the colony fold (NC/WTAP) were significantly decreased when treated with either SU9516 or K03861 respectively in CCK8 assay (Fig. 6c) and in the colony formation assay (Fig. 6d). Our result showed that the ability of proliferation was much fewer when CDK2 function was inhibited by inhibitors in Caki-1 and ACHN cells. Together, we would like to mention that the proliferation promoting induced by WTAP decreased when inhibition of the CDK2 function.

## Discussion

In this study, we firstly demonstrated that WTAP played an oncogenic role in RCC through binding to CDK2 transcript directly and enhancing its stability and was a prognostic indicator for RCC patients.

WTAP was found to be up-regulated both in RCC tissues and RCC cell lines. We also found WTAP expression was significantly positively correlated with tumor size and TNM stage by IHC analysis in RCC tissues. Furthermore, high WTAP expression was related to worse prognosis in RCC patients, implying that it can act as a predictor of overall survival in RCC patients. We further investigated the role of WTAP in the proliferation of RCC cells. CCK8 and colony formation assays showed that knockdown of WTAP could significantly decrease cell proliferation, while overexpression of WTAP had opposite effects. Further in vivo data finally supported the promoted function of WTAP in RCC cells. WTAP-knockdown RCC cells formed smaller tumor in nude mice compared to the control cells. These results were well consisted with experimental data both in vitro and in vivo. Xi et al. found that the WTAP was overexpressed in gliomas and its expression was significantly higher in high-grade glioma tissues [18]. WTAP was also overexpressed in cholangiocarcinoma and acute myeloid leukemia [15, 17]. All the results suggested that WTAP may play oncogenic roles in the tumorigenesis.

In cancer, proliferation is mostly driven by the altered cell cycle progression. The regulation of cell cycle is mainly dependent on cyclin and CDKs. Horiuchi et al. found WTAP could act as an essential factor for the stabilization of cyclin A2 mRNA, thereby regulating G2/M cell-cycle transition [23]. In this study, cell cycle arrest in G1 was observed in WTAP-knockdown RCC cells. We further found that CDK2 expression was obviously decreased in WTAP-knockdown RCC cells, whereas enhanced CDK2 expression occurred after WTAP overexpression in RCC cells. Moreover, IHC analysis showed a significant association between WTAP expression and CDK2 expression in RCC tissues. As an important regulatory factor in the cell cycle, CDK2 mainly acts on G1 phase and S phase, which is an important factor to initiate DNA replication [25]. Abnormal expression of CDK2 can make cell cross the G1/S limit and shorten the cell cycle and promote cell proliferation [22]. Abnormal expression of CDK2 was found in breast cancer [26], gastric cancer [27], colon cancer [28], and prostate cancer [29], which was closely related to the development of these tumors. Accordingly, CDK2 was also highly expressed in RCC and promoted its malignant proliferation [29]. Moreover, CDK2 knockdown by small interference RNA or CDK2 inhibitor totally reversed the cell growth promoting effects of WTAP in RCC cells. It implied that WTAP may play oncogenic roles in RCC mainly by the regulation of CDK2.

As a major component in human spliceosomes [10] and N6-methyladenosine methylation complex [30], WTAP was found to have the ability to regulate the targeted genes mRNA stability, acting as a RNA binding protein. In this study, WTAP was found to be able to increase CDK2 stability by extending its half-life after treated with actinomycin D for various times. Furthermore, RIP assays demonstrated that WTAP could bind to CDK2 transcript directly. Overexpression of WTAP significantly increased the luciferase activity of the CDK2 3′-UTR reporter vector, but not of the empty vector in dual-luciferase assay. All these results suggested WTAP could bind to CDK2 transcript directly in CDK2 3′-UTR and enhance its mRNA stability.

## Conclusions

In summary, our results showed that WTAP was up-regulated in RCC and could serve as a novel prognostic indicator for RCC patients. Moreover, WTAP significantly promoted RCC proliferation both in vitro and in vivo. WTAP could promote RCC cell proliferation by up-regulation of CDK2 through directly binding to its transcript and enhancing its mRNA stability.

## Additional files

Additional file 1: **Figure S1.** WTAP is a prognostic factor in RCC. The KM plot of tumor samples with detailed clinical information which were downloaded from TCGA database (https://cancergenome.nih.gov/). (TIFF 1834 kb)

Additional file 2: **Figure S2.** The efficiency of WTAP knockdown and overexpression in RCC cell lines. Caki-1 and ACHN cell were infected with WTAP overexpression lentivirus, a negative control, WTAP knockdown lentivirus, and a scramble control. The efficiency of WTAP knockdown **(A)** and overexpression **(B)** in Caki-1 and ACHN cell lines was screened by qRT-PCR and western blot. Data represent the mean ± SD from three independent experiments,*P < 0.05. (TIFF 3154 kb)

Additional file 3: **Figure S5.** Overexpression of WTAP could promote cell growth in HK-2 cell line. **(A)** The efficiency of WTAP overexpression in HK-2 cell lines was screened by western blot and qRT-PCR. **(B)** Proliferation of HK-2 cell with WTAP over-expressed assessed by CCK8 assays. Data represent the mean ± SD from three independent experiments, *P < 0.05. (TIFF 2677 kb)

Additional file 4: **Figure S3.** WTAP promotes RCC cell migration in vitro. Transwell migration**(A,B)** and the invasion capability **(C,D)** indicated that WTAP knockdown significantly decreased the number of cells crossing the membrane **(A, C)**, in contrast, cell migration and invasion were increased after overexpression of WTAP in both Caki-1 and ACHN cell lines **(B, D)**. Data represent the mean ± SD from three independent experiments,*P < 0.05. (TIFF 10013 kb)

Additional file 5: **Figure S4.** The expression of WTAP and CDK2 was positively correlated in RCC tissues. A scatter plot of WTAP and CDK2 relative expression in the tumor samples which were downloaded from TCGA database (https://cancergenome.nih.gov/). (2-tailed Spearman’s correction, *R* = 0.1604, *P* = 0.0039) (TIFF 3836 kb)

Additional file 6: **Figure S6.** WTAP regulated cyclin A2 expression in RCC cells and correlated with cyclin A2 expression in human RCC tissues. **(A)** Western blot analysis of cyclin A2 expression in Caki-1 cells with WTAP knockdown or overexpression. Cyclin A2 expression was obviously decreased in WTAP-knockdown cells whereas increased in WTAP overexpression cells. **(B)** The expression of WTAP and cyclin A2 was positively correlated in RCC tissues. A scatter plot of WTAP and cyclin A2 relative expression in the tumor samples which were downloaded from TCGA database (https://cancergenome.nih.gov/) (2-tailed Spearman’s correction, *R* = 0.25, *P* = 1.3e-12). **(C)** WTAP knockdown or overexpression cells were treated with actinomyclin D (Act D). Total RNAs were harvested, and then subjected to quantitative RT-PCR analysis. Knockdown of WTAP could shorten the half-life of cyclin A2 transcript. While, ectopic expression of WTAP could longthen the half-life of cylcin A2 transcript. Data represent the mean ± SD from three independent experiments,*P < 0.05. (TIFF 938 kb)

Additional file 7: **Figure S7.** WTAP enhanced the stability of the CDK2 transcript. **(A)** Knockdown of WTAP could shorten the half-life of CDK2 transcript. Cells were treated with 5μg/ml actinomyclin D (Act D) and performed the qRT-PCR. GADPH was used as another stable reference mRNA. The relative quantification was calculated by the 2^−ΔΔCt^ method and normalized based on GADPH. **(B)** Ectopic expression of WTAP could longthen the half-life of CDK2 transcript. Data represent the mean ± SD from three independent experiments,*P < 0.05. (TIFF 604 kb)

## Abbreviations

3′-UTR: 3′-untranslated region
Act D: Actinomyclin D
CDK: Cyclin-dependent protein kinase
IHC: Immunohistochemistry
RCC: Renal cell carcinoma
RIP: RNA immunoprecipitation
siRNA: small interfering RNA
WTAP: Wilms′ tumor 1-associating protein
