# Supplementary figures and images for: Wilms’ tumor 1-associating protein promotes renal cell carcinoma proliferation by regulating CDK2 mRNA stability
Source: J Exp Clin Cancer Res. 2018 Feb 27;37:40. doi: 10.1186/s13046-018-0706-6 (PMC5827993; doi:10.1186/s13046-018-0706-6)

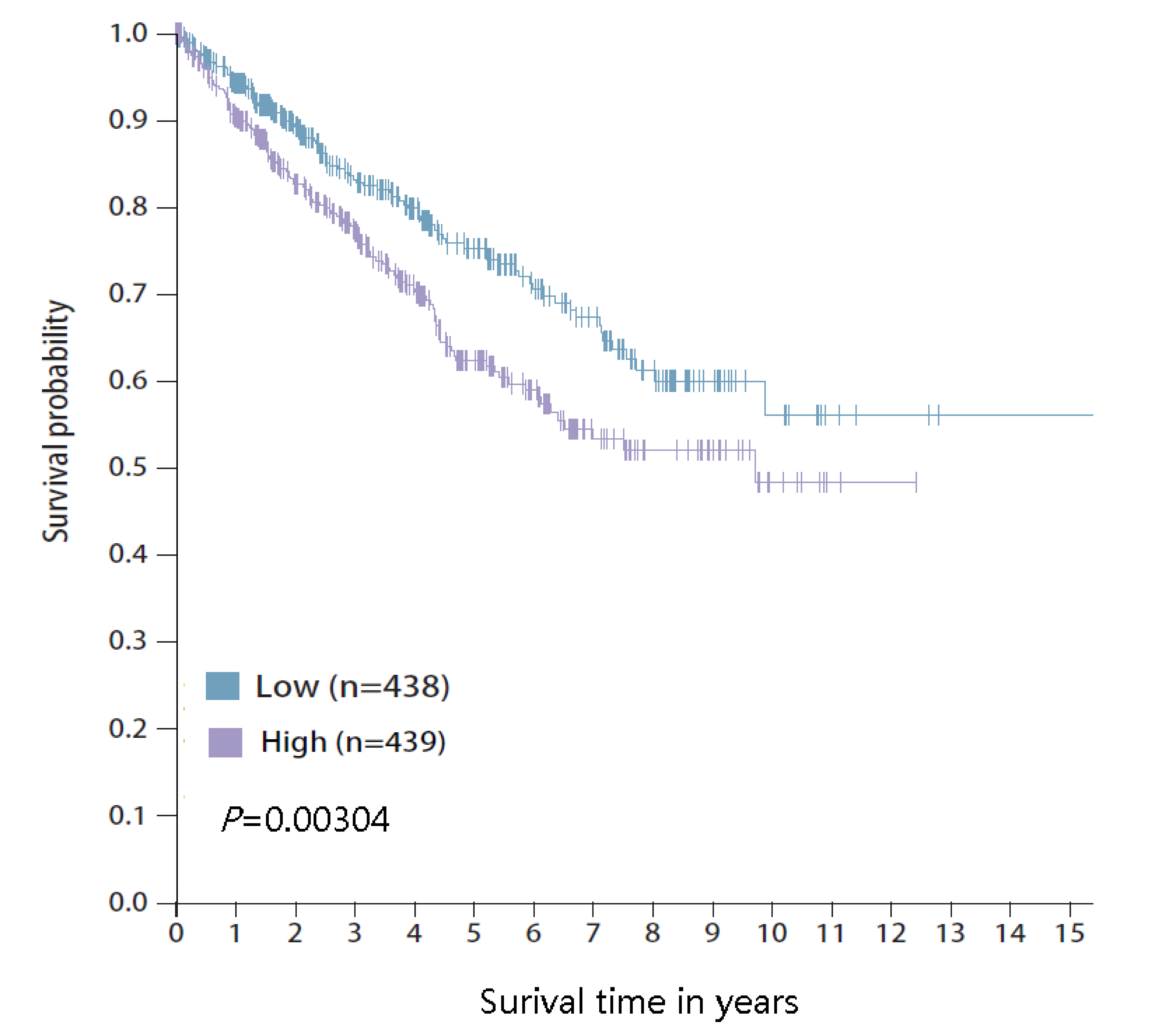

Supplement: Supplementary file 1 — Figure S1. WTAP is a prognostic factor in RCC. The KM plot of tumor samples with detailed clinical information which were downloaded from TCGA database (https://cancergenome.nih.gov/). (TIFF 1834 kb) [file 13046_2018_706_MOESM1_ESM.tif]

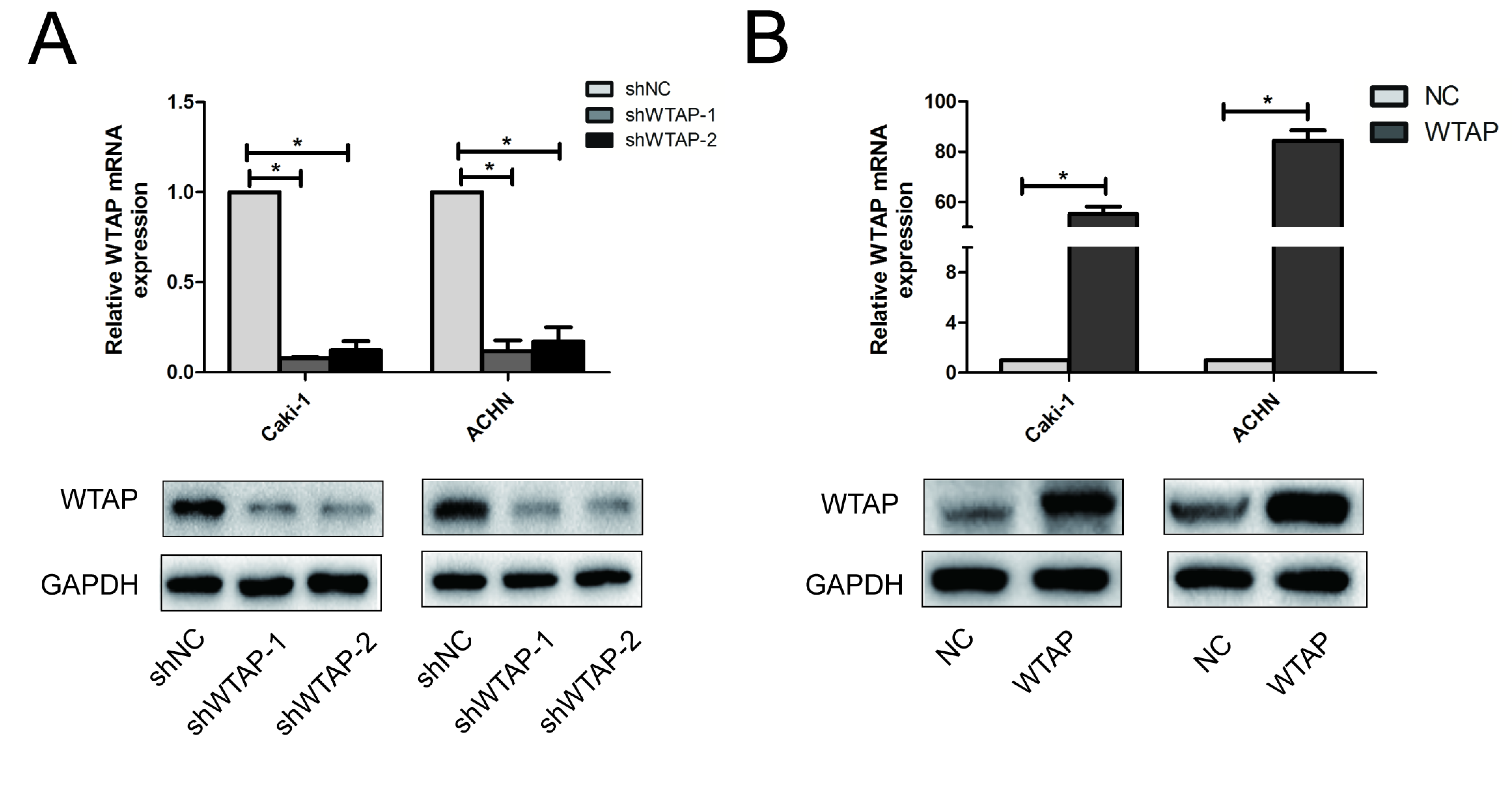

Supplement: Supplementary file 2 — Figure S2. The efficiency of WTAP knockdown and overexpression in RCC cell lines. Caki-1 and ACHN cell were infected with WTAP overexpression lentivirus, a negative control, WTAP knockdown lentivirus, and a scramble control. The efficiency of WTAP knockdown (A) and overexpression (B) in Caki-1 and ACHN cell lines was screened by qRT-PCR and western blot. Data represent the mean ± SD from three independent experiments,*P < 0.05. (TIFF 3154 kb) [file 13046_2018_706_MOESM2_ESM.tif]

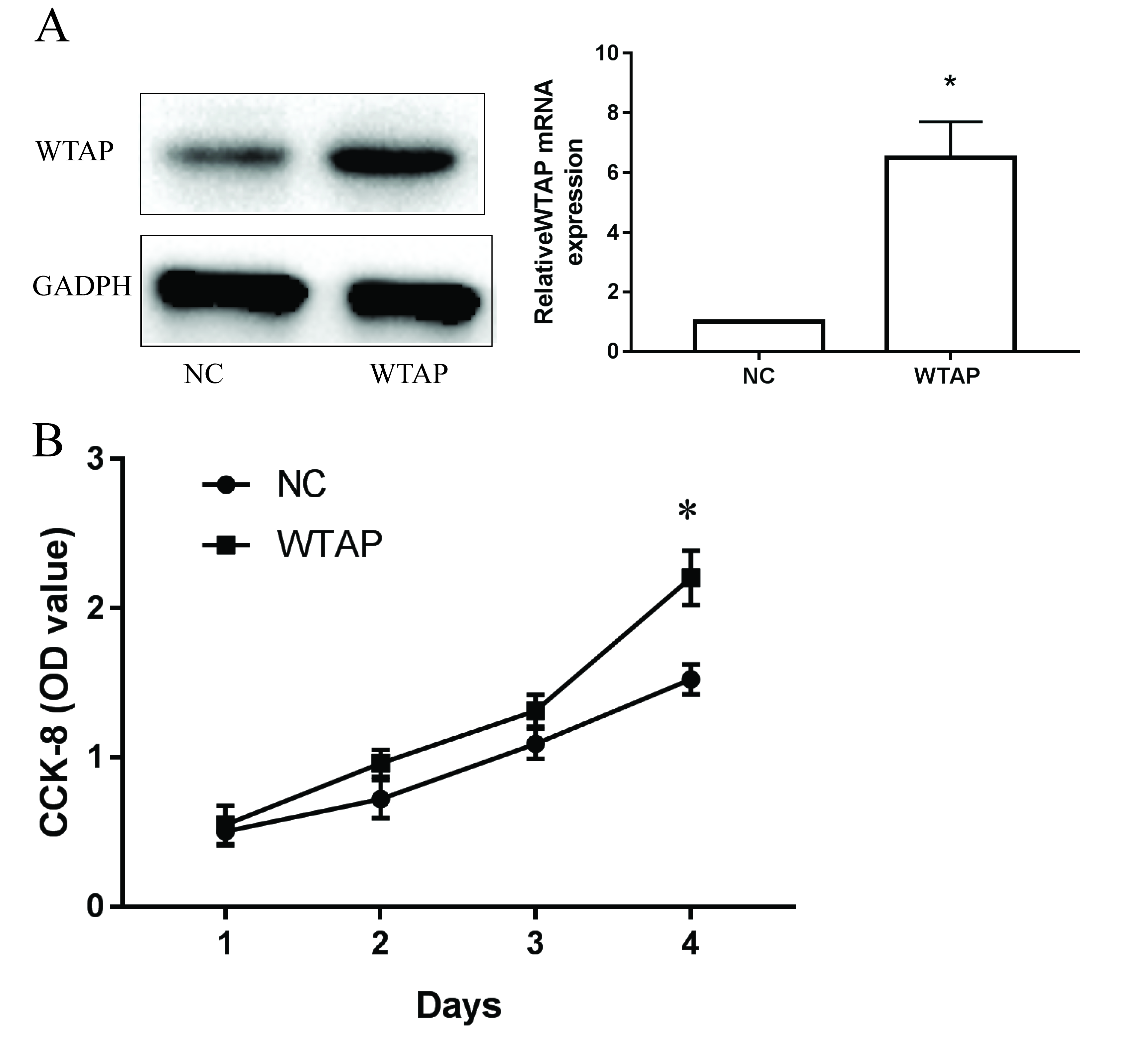

Supplement: Supplementary file 3 — Figure S5. Overexpression of WTAP could promote cell growth in HK-2 cell line. (A) The efficiency of WTAP overexpression in HK-2 cell lines was screened by western blot and qRT-PCR. (B) Proliferation of HK-2 cell with WTAP over-expressed assessed by CCK8 assays. Data represent the mean ± SD from three independent experiments, *P < 0.05. (TIFF 2677 kb) [file 13046_2018_706_MOESM5_ESM.tif]

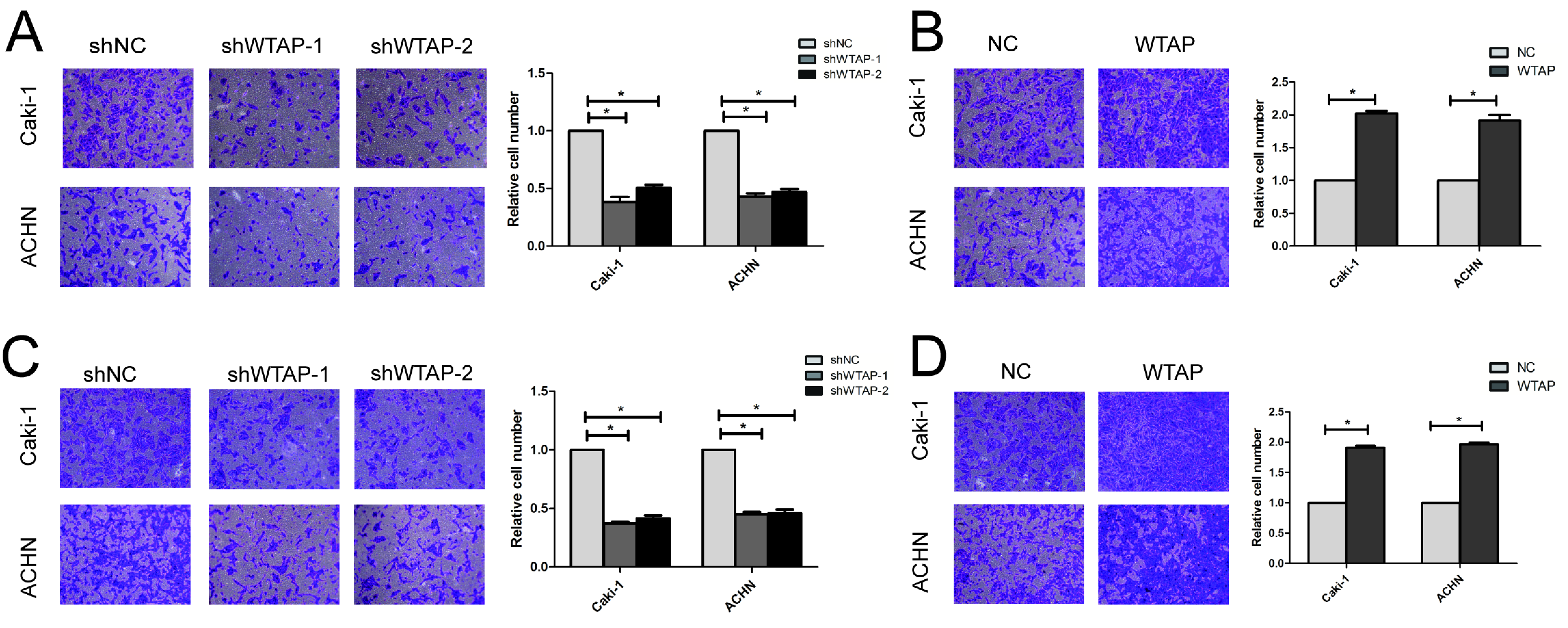

Supplement: Supplementary file 4 — Figure S3. WTAP promotes RCC cell migration in vitro. Transwell migration(A,B) and the invasion capability (C,D) indicated that WTAP knockdown significantly decreased the number of cells crossing the membrane (A, C), in contrast, cell migration and invasion were increased after overexpression of WTAP in both Caki-1 and ACHN cell lines (B, D). Data represent the mean ± SD from three independent experiments,*P < 0.05. (TIFF 10013 kb) [file 13046_2018_706_MOESM3_ESM.tif]

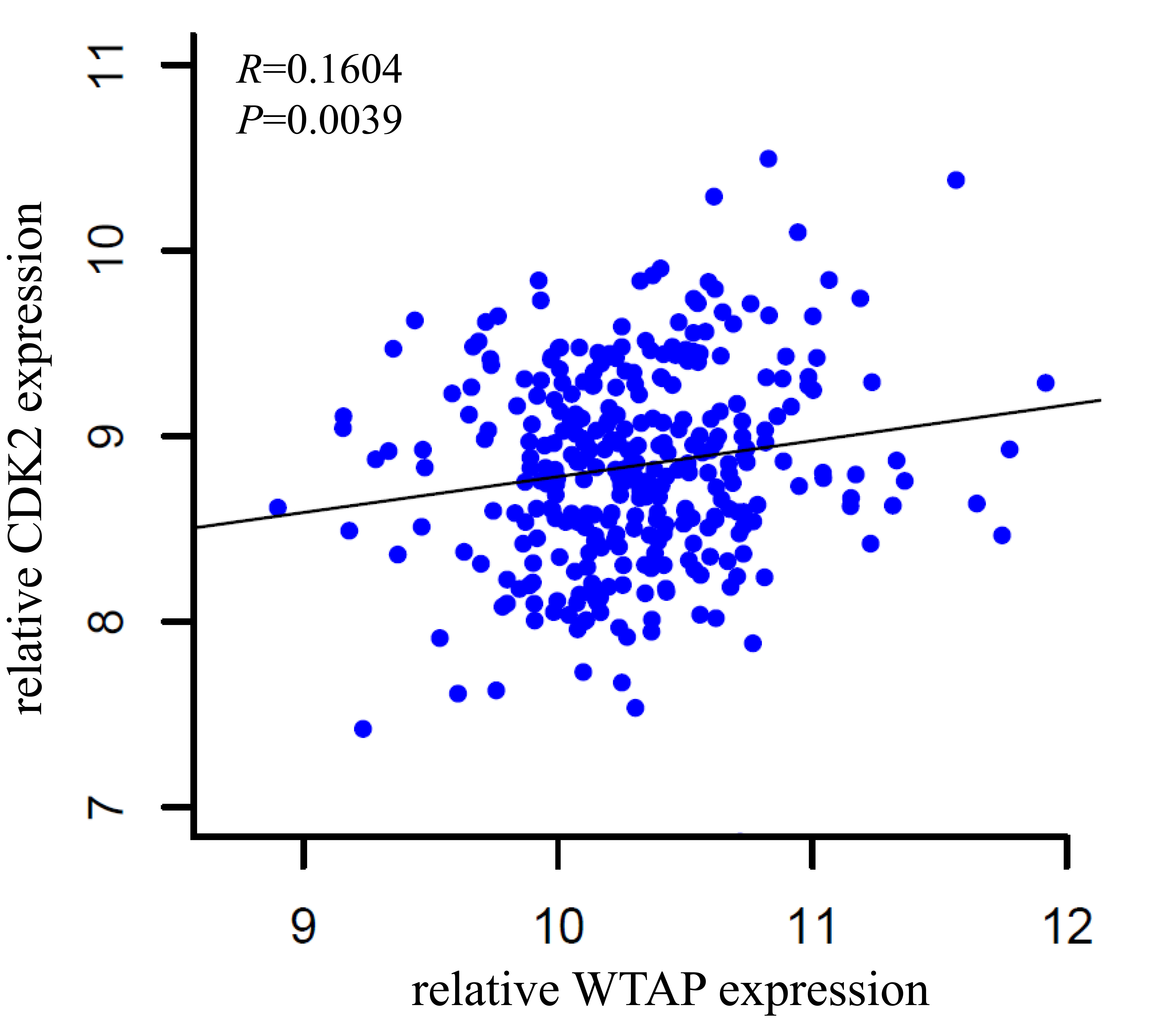

Supplement: Supplementary file 5 — Figure S4. The expression of WTAP and CDK2 was positively correlated in RCC tissues. A scatter plot of WTAP and CDK2 relative expression in the tumor samples which were downloaded from TCGA database (https://cancergenome.nih.gov/). (2-tailed Spearman’s correction, R = 0.1604, P = 0.0039) (TIFF 3836 kb) [file 13046_2018_706_MOESM4_ESM.tif]

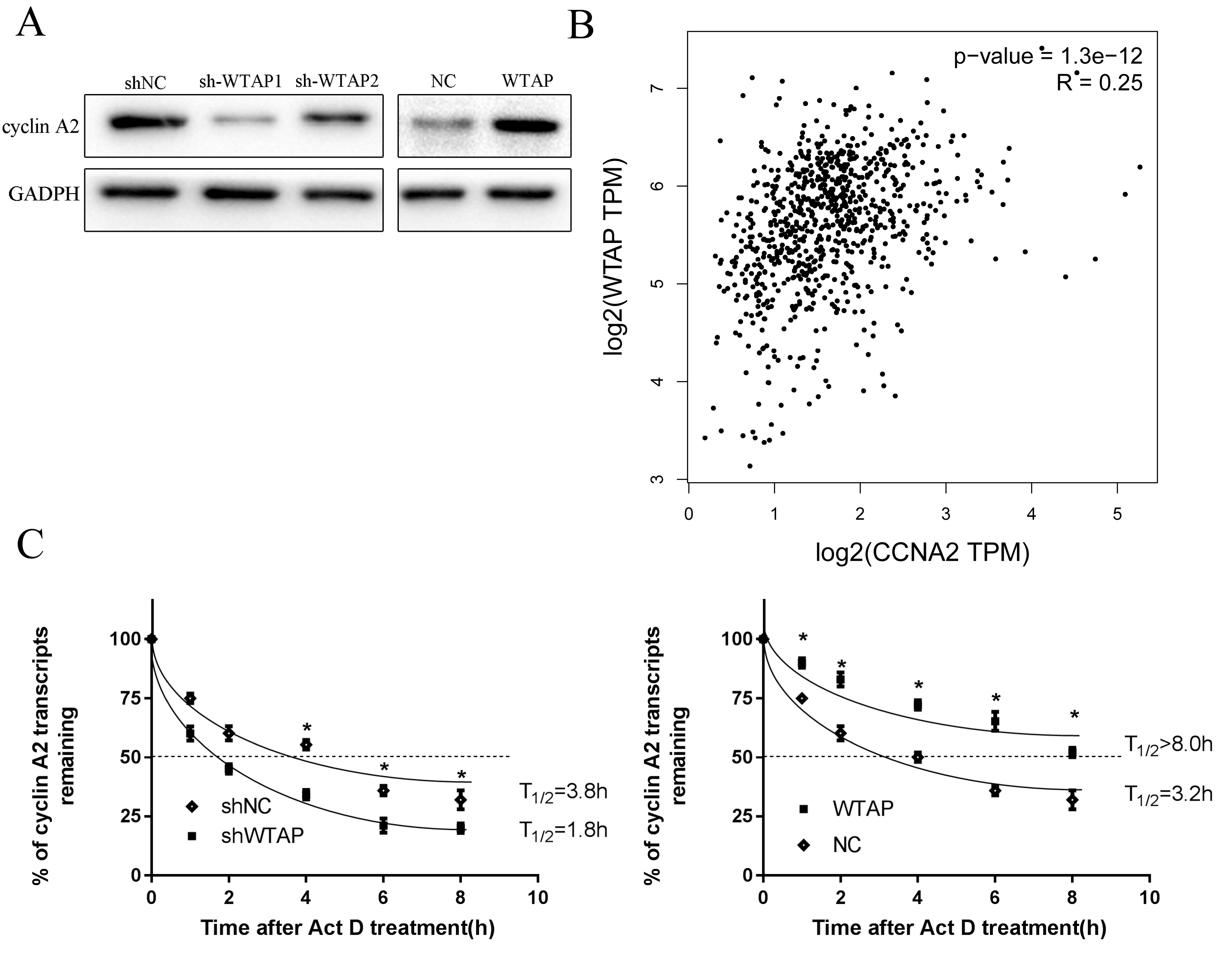

Supplement: Supplementary file 6 — Figure S6. WTAP regulated cyclin A2 expression in RCC cells and correlated with cyclin A2 expression in human RCC tissues. (A) Western blot analysis of cyclin A2 expression in Caki-1 cells with WTAP knockdown or overexpression. Cyclin A2 expression was obviously decreased in WTAP-knockdown cells whereas increased in WTAP overexpression cells. (B) The expression of WTAP and cyclin A2 was positively correlated in RCC tissues. A scatter plot of WTAP and cyclin A2 relative expression in the tumor samples which were downloaded from TCGA database (https://cancergenome.nih.gov/) (2-tailed Spearman’s correction, R = 0.25, P = 1.3e-12). (C) WTAP knockdown or overexpression cells were treated with actinomyclin D (Act D). Total RNAs were harvested, and then subjected to quantitative RT-PCR analysis. Knockdown of WTAP could shorten the half-life of cyclin A2 transcript. While, ectopic expression of WTAP could longthen the half-life of cylcin A2 transcript. Data represent the mean ± SD from three independent experiments,*P < 0.05. (TIFF 938 kb) [file 13046_2018_706_MOESM6_ESM.tif]

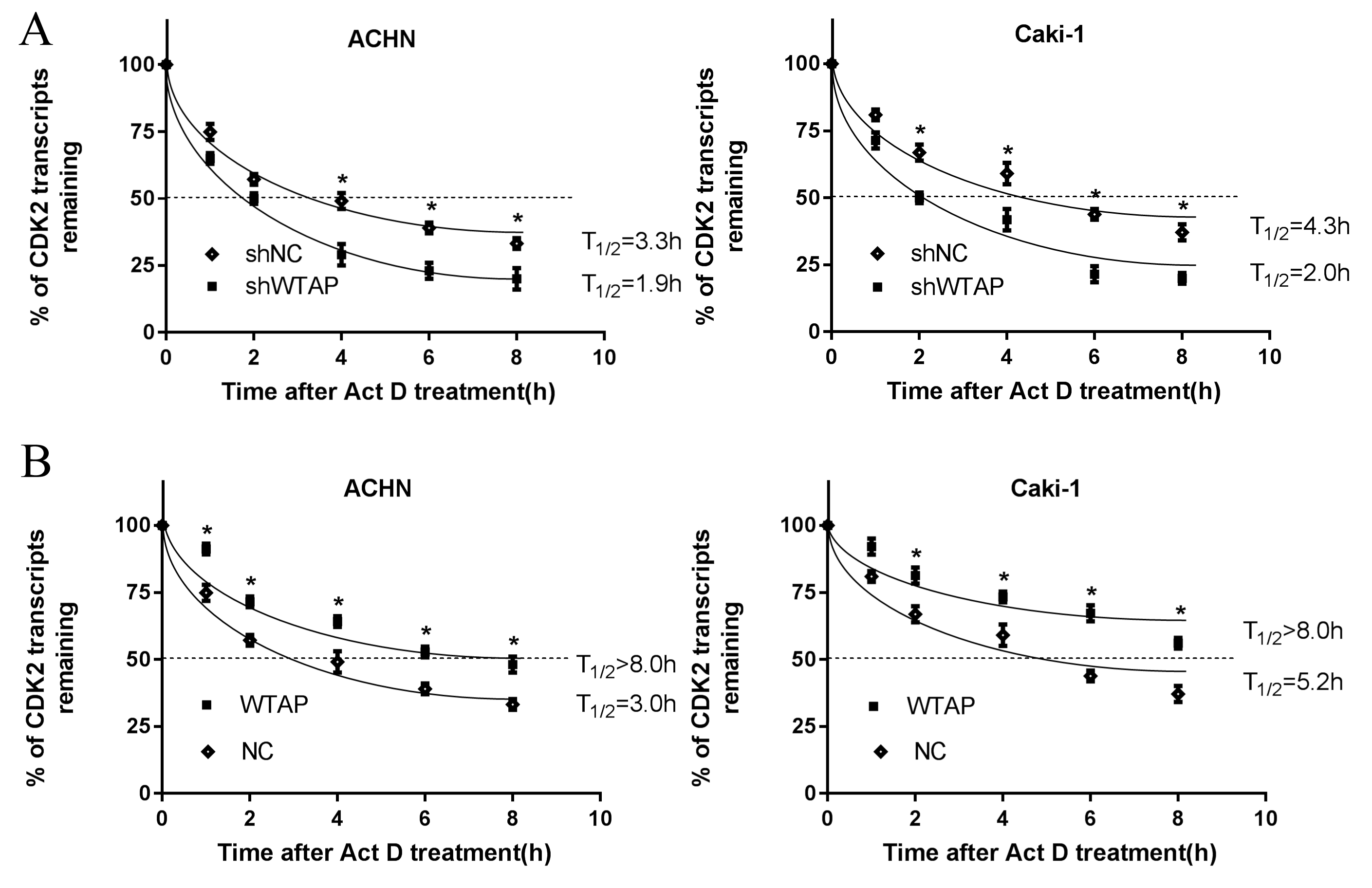

Supplement: Supplementary file 7 — Figure S7. WTAP enhanced the stability of the CDK2 transcript. (A) Knockdown of WTAP could shorten the half-life of CDK2 transcript. Cells were treated with 5μg/ml actinomyclin D (Act D) and performed the qRT-PCR. GADPH was used as another stable reference mRNA. The relative quantification was calculated by the 2−ΔΔCt method and normalized based on GADPH. (B) Ectopic expression of WTAP could longthen the half-life of CDK2 transcript. Data represent the mean ± SD from three independent experiments,*P < 0.05. (TIFF 604 kb) [file 13046_2018_706_MOESM7_ESM.tif]
